# Neurological disorders associated with COVID-19 in Sri Lanka

**DOI:** 10.1186/s12883-023-03399-w

**Published:** 2023-10-04

**Authors:** Thashi Chang, Ruwani Wijeyekoon, Ajantha Keshavaraj, Udaya Ranawaka, Sunethra Senanayake, Pyara Ratnayake, Bimsara Senanayake, Manjula C. Caldera, Gamini Pathirana, Darshana Sirisena, Jithangi Wanigasinghe, Saman Gunatilake, on behalf of the ASN COVID-19 Study Group, A. Keshavaraj, U. K. Ranawaka, S. Senanayake, P. Ratnayake, B. Senanayake, M. C. Caldera, D. Halahakoon, D. S. Wijesekara, S. Bandusena, T. Chang, H. Gunasekara, C. Gunawardhana, A. Arasalingam, A. Fernando, D. S. Liyanage, G. Pathirana, T. N. P. Rathnayake, A. T. Alibhoy, D. N. Weerathunga, A. Dissanayake, K. Gooneratne, A. Jayawardana, T. Nawasiwatte, V. T. Rajendiran, D. Rathnayake, J. Wanigasinghe, G. J. Arhivalaky, S. Branavan, M. J. N. Fernando, K. Janarthanan, K. Kariyawasam, N. I. Karunasena, D. Luke, M. K. T. Madhushanka, S. N. H. Nimesha, M. P. Priyacharana, T. D. Ruvanpathirana, DPUT Samarasiri, S. C. Weerasinghe

**Affiliations:** 1 Department of Clinical Medicine, Faculty of Medicine, University of Colombo, Colombo, Sri Lanka; 2 Association of Sri Lankan Neurologists, Wijerama Mawatha, Colombo, Sri Lanka; 3 Teaching Hospital Jaffna, Jaffna, Sri Lanka; 4 Department of Medicine, Faculty of Medicine, University of Kelaniya, Kelaniya, Sri Lanka; 5 National Hospital of Sri Lanka, Colombo, Sri Lanka; 6 Lady Ridgeway Hospital for Children, Colombo, Sri Lanka; 7 Teaching Hospital Anuradhapura, Anuradhapura, Sri Lanka; 8 Colombo North Teaching Hospital, Ragama, Sri Lanka; 9 Department of Paediatrics, Faculty of Medicine, University of Colombo, Colombo, Sri Lanka; 10 Department of Medicine, University of Sri Jayewardenepura, Nugegoda, Sri Lanka

**Keywords:** COVID-19, SARS-CoV-2, Neurology, Sri Lanka

## Abstract

**Background:**

Neurological manifestations of SARS-CoV-2 infection have been reported from many countries around the world, including the South Asian region. This surveillance study aimed to describe the spectrum of neurological disorders associated with COVID-19 in Sri Lanka.

**Methods:**

COVID-19 patients manifesting neurological disorders one week prior and up to six weeks after infection were recruited from all the neurology centres of the government hospitals in Sri Lanka from May 2021 – May 2022. Data was collected using a structured data form that was electronically transmitted to a central repository. All patients were evaluated and managed by a neurologist. Data were analysed using simple descriptive analysis to characterise demographic and disease related variables, and simple comparisons and logistic regression were performed to analyse outcomes and their associations.

**Results:**

One hundred and eighty-four patients with neurological manifestations associated with COVID-19 were recruited from all nine provinces in Sri Lanka. Ischaemic stroke (31%) was the commonest neurological manifestation followed by encephalopathy (13.6%), Guillain–Barre syndrome (GBS) (9.2%) and encephalitis (7.6%). Ischaemic stroke, encephalitis and encephalopathy presented within 6 days of onset of COVID-19 symptoms, whereas GBS and myelitis presented up to 10 days post onset while epilepsy and Bell palsy presented up to 20 – 40 days post onset. Haemorrhagic stroke presented either just prior to or at onset, or 10 – 25 days post onset of COVID-19 symptomatic infection. An increased frequency of children presenting with encephalitis and encephalopathy was observed during the Omicron variant predominant period.

A poor outcome (no recovery or death) was associated with supplemental oxygen requirement during admission (Odds Ratio: 12.94; *p* = 0.046).

**Conclusions:**

The spectrum and frequencies of COVID-19 associated neurological disorders in Sri Lanka were similar to that reported from other countries, with strokes and encephalopathy being the commonest. Requiring supplemental oxygen during hospitalisation was associated with a poor outcome.

**Supplementary Information:**

The online version contains supplementary material available at 10.1186/s12883-023-03399-w.

## Background

The coronavirus disease 2019 (COVID-19) pandemic caused by the novel coronavirus, severe acute respiratory syndrome coronavirus 2 (SARS-CoV-2), has to date (November 2022) led to over 633,000,000 cases and over 6,500,000 deaths worldwide [1]. An increasing number of patients have been reported to develop a variety of neurological disorders associated with COVID-19 infection [2, 3]. These neurological manifestations are considered to be related to vascular, inflammatory and infective aetiologies, and include both ischaemic and haemorrhagic stroke, cerebral venous thrombosis, encephalitis/encephalopathy, acute disseminated encephalomyelitis (ADEM), Guillain–Barre syndrome (GBS), myopathy and olfactory dysfunction [4–6].

Sri Lanka detected its first case of COVID-19 in March 2020 and has since had over 600,000 cases and over 16,000 deaths (November 2022) [7]. This is the first systematic study of neurological disorders associated with the COVID-19 pandemic in Sri Lanka.

## Methods

The Association of Sri Lankan Neurologists (ASN), which is the apex professional body for neurologists in Sri Lanka, set up a neurological surveillance study of COVID-19 in May 2021 with the aim of recruiting all patients with neurological disorders associated with COVID-19 from government and private hospitals with access to services of a specialist neurologist. The study was done over a period of 12 months. Government hospitals are non-fee-levying and are accessible by all. Neurologists are usually available in tertiary care level hospitals and such hospitals are situated in all nine administrative provinces in the country. Patients with neurological disorders associated with COVID-19 admitted to smaller hospitals usually are referred to hospitals with neurology centres.

All patients developing neurological manifestations one week prior, and up to six weeks after, diagnosis of COVID-19 were included in the study. Patients in whom neurological features were confirmed to be related to another diagnosis were excluded. All patients were under the care of the attending neurologist who screened the patient for eligibility into the study. Patient data were then collected by completing a structured data form developed by an expert committee of the ASN (Additional file 1). Data were collected on neurological features, COVID-19 symptoms and signs, treatment given, need for intensive care and ventilation, outcome and vaccination status, and were transmitted electronically to a central repository. Definitions of the neurological syndromes were provided in the data form in order to ensure consistency [2]. In cases of ambiguity as to whether the neurological manifestation was related to COVID-19 or incidental, a consensus decision was made after independent review by a second neurologist in addition to the attending neurologist.

Ethics clearance for the study was obtained from the Ethics Review Committee (ERC) of the University of Colombo (EC-21–070). All methods were carried out in accordance with relevant guidelines and regulations of the ERC. Informed consent was obtained from all subjects and/or their legal guardian(s).

Data were analysed using SPSS version 27 and Graph Pad Prism V9. Mean and standard deviation (SD) or percentage values for demographic, clinical and laboratory variables were calculated. Associations between demographic, clinical or investigation variables and neurological manifestations and outcomes were investigated. Logistic regression analysis was used to investigate factors associated with a relatively better outcome (complete/ partial recovery) versus a poor outcome (no recovery/ death). Significance was determined at *p* < 0.05.

## Results

### Patient cohort and demographics

**Fig. 1:**
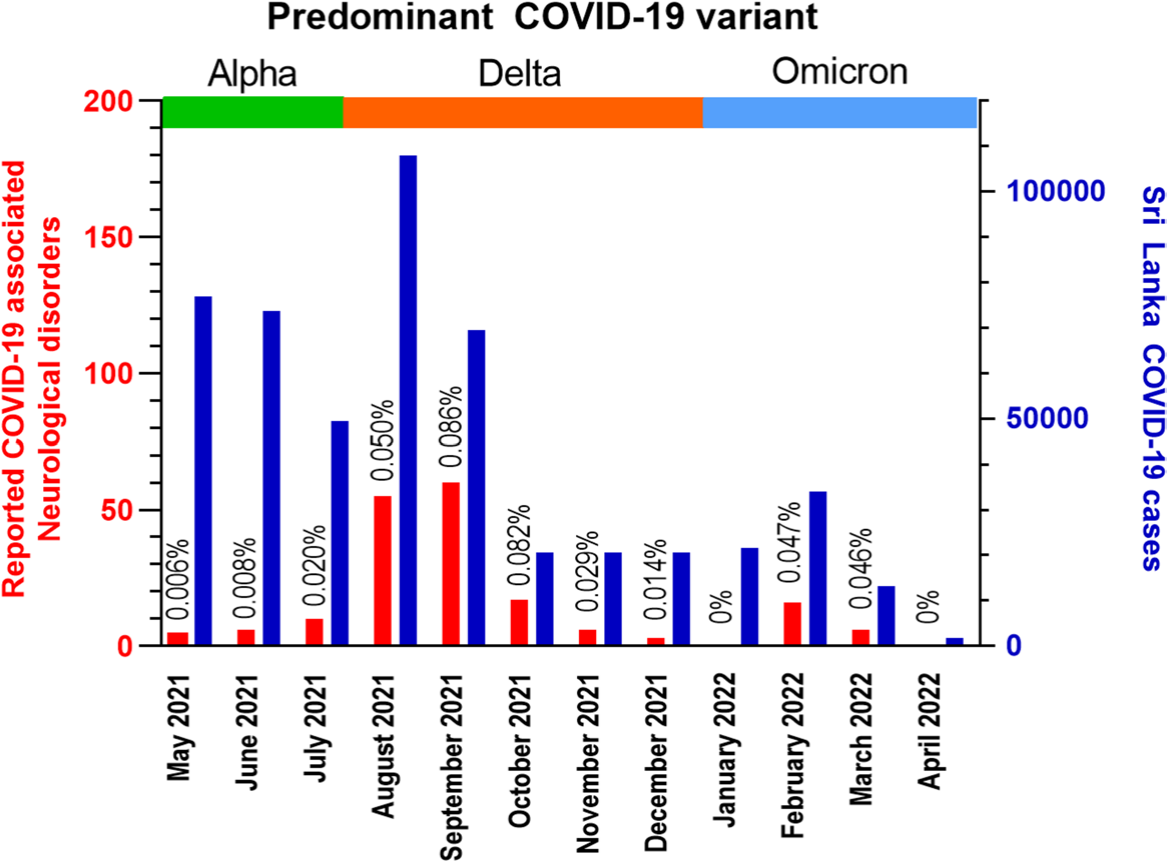
Monthly frequencies of reported COVID-19 associated neurological disorders (red – left Y axis) relative to monthly nationally reported COVID-19 cases in Sri Lanka (blue – right Y axis). Percentages indicate monthly reported COVID-19 associated neurological disorders as a percentage of monthly nationally reported COVID-19 cases. Horizontal bars indicate predominant COVID-19 variant reported to be present in Sri Lanka during the relevant periods

One hundred and eighty-four patients were recruited within the study period of 12 months. The highest number of patients were reported from the administrative districts of Jaffna, Colombo and Gampaha, of which the latter two are the most populace districts in Sri Lanka. The frequency of COVID-19 associated neurological disorders reported per month peaked at 60 cases/month in September 2021 following a peak in nationally reported COVID-19 cases in August 2021 (Fig. 1). Based on samples sequenced from one laboratory, the predominant COVID-19 variant in Sri Lanka transitioned from Alpha to Delta around July/August 2021 and from Delta to Omicron in January 2022 [8]. The majority of patients (76.6%) were reported during the time period of predominance of the Delta variant AY.28 [9].

SARS-CoV-2 infection was confirmed in the majority of patients by polymerase chain reaction (PCR) testing (51.6%) or rapid antigen testing (RAT) (46.7%) of throat/nasal swabs. A small number of patients were diagnosed using the presence of antibodies in serum (0.54%) or cerebrospinal fluid (CSF) (1.08%) in the absence of a history of vaccination for COVID-19.

Demographic and clinical details of the cohort are shown in Table 1. The mean age of the study population was 50.6 years (range 1 – 89 years); 54.3% were males. The most common co-morbidities were hypertension (29.3%), diabetes mellitus (25.5%) and ischaemic heart disease (16%) (Table 1).

**Table 1:** Demographic and co-morbidity characteristics, COVID-19 infection related features, key management requirements and COVID-19 vaccination status of the study cohort. Bold text indicates subsection headings. (ICU - intensive care unit, SD - standard deviation)

**Characteristic**	
Mean age (SD, range), years	50.6 (23.4, 1–89)
	**Number (%),** ***N*** **= 184**
Males	100 (54.3)
Smoking	11 (6.0)
Alcohol excess	11 (6.0)
Pregnant	3 (1.6)
Immunosuppressant medication	2 (1.1)
**Comorbidity**	**Number (%),** ***N*** **= 184**
Hypertension	54 (29.3)
Diabetes	47 (25.5)
Ischaemic heart disease	16 (8.7)
Chronic kidney disease	12 (6.5)
Obesity	11 (6.0)
Chronic pulmonary disease	9 (4.9)
Dementia	8 (4.3)
Dyslipidaemia	5 (2.7)
Malignancy	2 (1.1)
Chronic liver disease	2 (1.1)
Cardiac failure	1 (0.5)
Other (craniopharyngioma, subarachnoid haemorrhage and dural arteriovenous fistula, mitral stenosis and pulmonary hypertension, epilepsy, renal transplant, gall bladder disease, thalassaemia, anaemia, Parkinson’s disease, mumps orchitis, venous ulcers, autoimmune encephalitis, rheumatoid arthritis)	16 (8.7)
No comorbidity	56 (30.4)
**COVID-19 infection related features**	**Number (%),** ***N*** **= 176**
Symptomatic	130 (73.9)
**Features within symptomatic patients**	**Number (%),** ***N*** **= 130**
COVID pneumonia	61 (46.9)
Anosmia	26 (20.0)
Sepsis	18 (13.8)
Ageusia	10 (7.6)
Deep vein thrombosis/Pulmonary embolism	1 (0.8)
**COVID-19 infection management requirements**	**Number (%),** ***N*** **= 184**
Supplemental oxygen	52 (28.3)
ICU Care	12 (6.5)
Non-invasive ventilation	11 (6.0)
Mechanical ventilation	10 (5.4)
**COVID-19 vaccination status**	**Number (%),** *N* **= 184**
Unvaccinated	96 (52.2)
Vaccinated—1 dose	33 (17.9)
Vaccinated—2 doses	54 (29.3)
Vaccinated—3 doses	1 (0.5)

Seventy four percent of the cohort had symptoms of COVID-19, of which 47% had COVID-19 pneumonia (Table 1). Twenty eight percent of the overall cohort required supplemental oxygen, while only 6.5% of patients required intensive care unit (ICU) admission and assisted ventilation (Table 1).

Nearly 48% of the cohort had received at least one COVID-19 vaccination, with 17.9% having had one dose, 29.3% two doses and 0.5% three doses (Table 1). The majority (89.8%) of patients had received the Sinopharm inactivated COVID-19 vaccine (Vero cell) manufactured by the Beijing Institute of Biological Products Co., Limited (BIBP).

### Neurological disorders associated with COVID-19

**Fig. 2:**
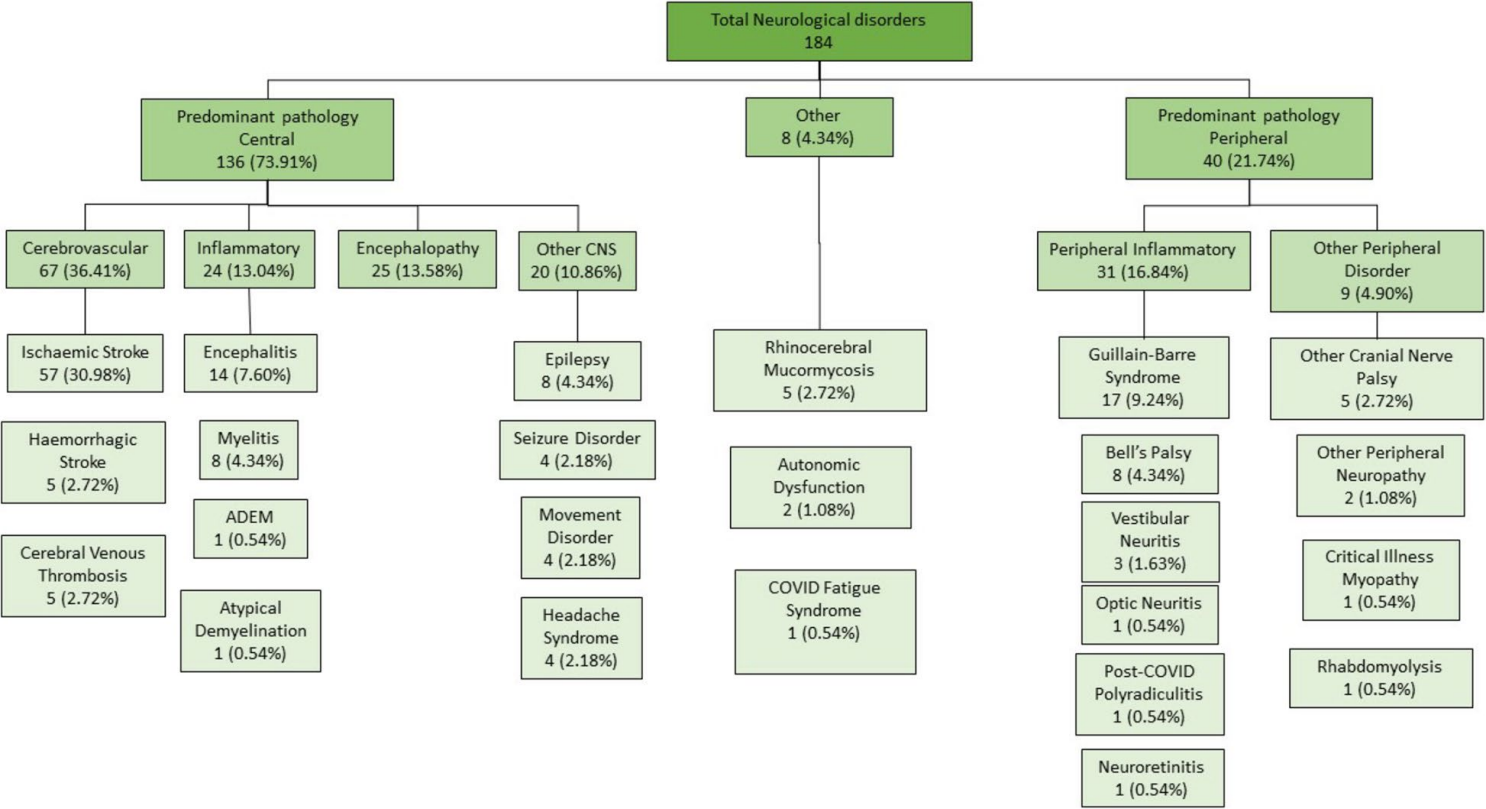
Classification of main neurological diagnoses reported as associated with COVID-19. (ADEM—acute disseminated encephalomyelitis; CNS—central nervous system)

The majority of the neurological disorders associated with COVID-19 affected the central nervous system (CNS) (*n* = 136; 73.91%). In 40 patients (21.74%) the predominant pathology was peripheral, while in 8 patients (4.34%) the disorders could not be classified as central or peripheral and thus, were classified as ‘other’ (Fig. 2). Of the CNS disorders, cerebrovascular pathology was the most common. Encephalitis and myelitis were the most frequent CNS inflammatory disorders (Fig. 2). Of the 40 patients with peripheral disorders, 31 (16.84%) were classed as having an inflammatory basis and included 17 (9.24%) with GBS, eight (4.34%) with Bell palsy, three (1.63%) with vestibular neuritis, and one (0.54%) each with optic neuritis, post-COVID polyradiculitis and neuroretinitis (Fig. 2). Conditions in the ‘other’ category included COVID fatigue syndrome, autonomic dysfunction and rhinocerebral mucormycoses (Fig. 2).

**Fig. 3:**
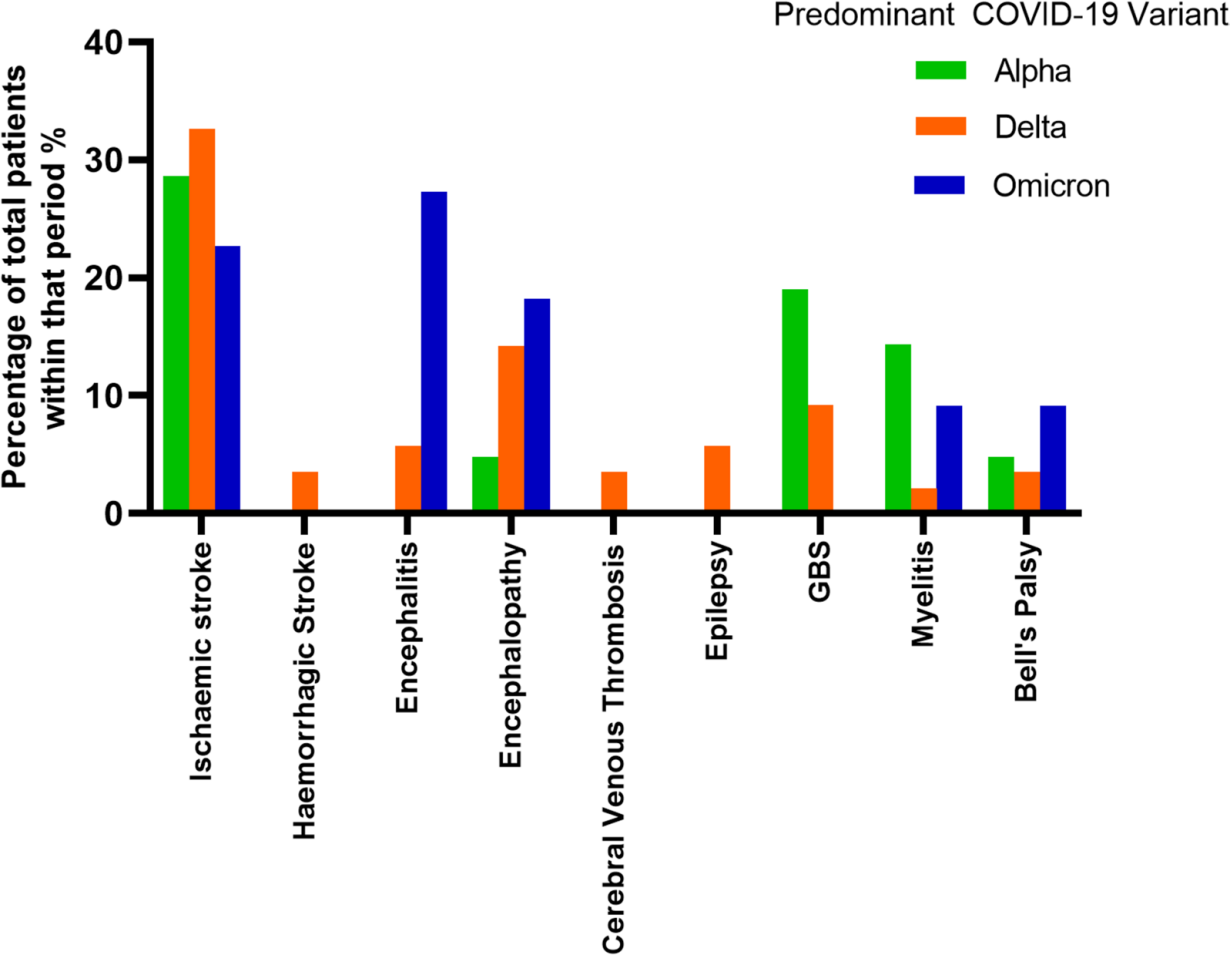
Spectrum of neurological disorders in relation to the predominant COVID-19 variant present at the time in Sri Lanka

The pattern and spectrum of reported neurological disorders appeared to be related to the predominant type of COVID-19 variant (Fig. 3). Ischaemic stroke, GBS and myelitis were the dominant conditions seen during the Alpha variant predominant period, while ischaemic stroke, encephalopathy and GBS were the dominant conditions in the Delta variant predominant period. In contrast, encephalitis was the dominant disorder in the Omicron variant predominant period, followed by ischaemic stroke, encephalopathy, myelitis and Bell palsy (Fig. 3). The average age of the patients presenting in the Omicron predominant period was significantly lower than the other periods (Mean years (SD) – Alpha 43.9 (19.0), Delta 55.6 (21.0), Omicron 24.9 (23.9); between groups analysis of variance (ANOVA): F = 21.2, *p* < 0.001), with 50% of patients reported in the Omicron predominant period being 0–10 years in age, including 4 patients with encephalitis and 4 with encephalopathy.

Three pregnant patients were reported due to haemorrhagic stroke, encephalitis and epilepsy. Among the 22 paediatric patients reported (< 18 years), the predominant neurological conditions included encephalitis (27.3%), encephalopathy (18.2%), GBS (13.6%) and myelitis (9.1%).

### Timing of onset of neurological disorders in relation to COVID-19

**Fig. 4:**
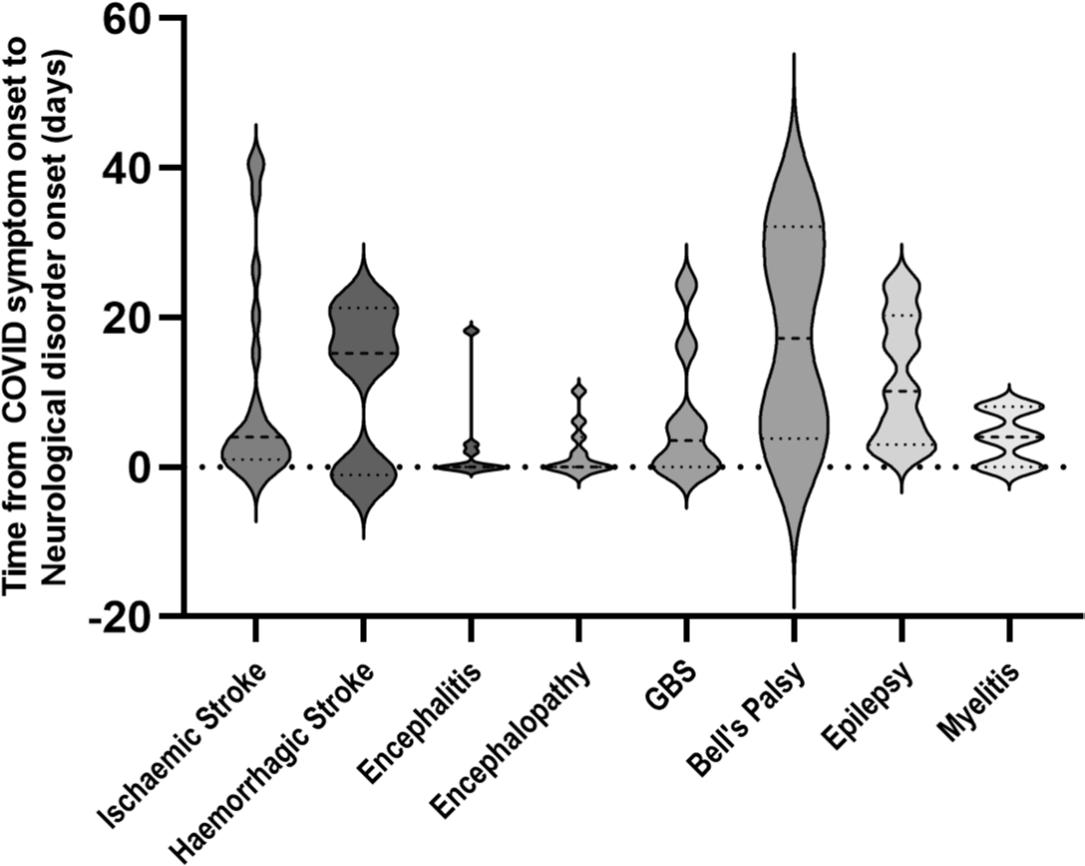
Violin plot showing time of onset of neurological disorders in relation to the onset of COVID-19 symptoms denoted as time point 0 day. (GBS: Guillain–Barre syndrome)

The timing of the onset of neurological symptoms in relation to the onset of symptomatic COVID-19 for some of the key conditions is shown in Fig. 4. The onset of ischaemic stroke, encephalitis, encephalopathy, were generally < 5–6 days from onset of typical COVID-19 symptoms, while GBS and myelitis occurred mostly less than 10 days after onset of symptoms. Haemorrhagic strokes presented at two time points, either at/just prior to the onset of symptomatic COVID-19 or later at 10–25 days post-COVID-19 symptom onset. Epilepsy presented more frequently from early on, until about 20 days after the onset of COVID-19 symptoms while Bell palsy had a wider window from COVID symptom onset up to 40 days later (Fig. 4).

### Outcomes of neurological disorders associated with COVID-19

**Fig. 5:**
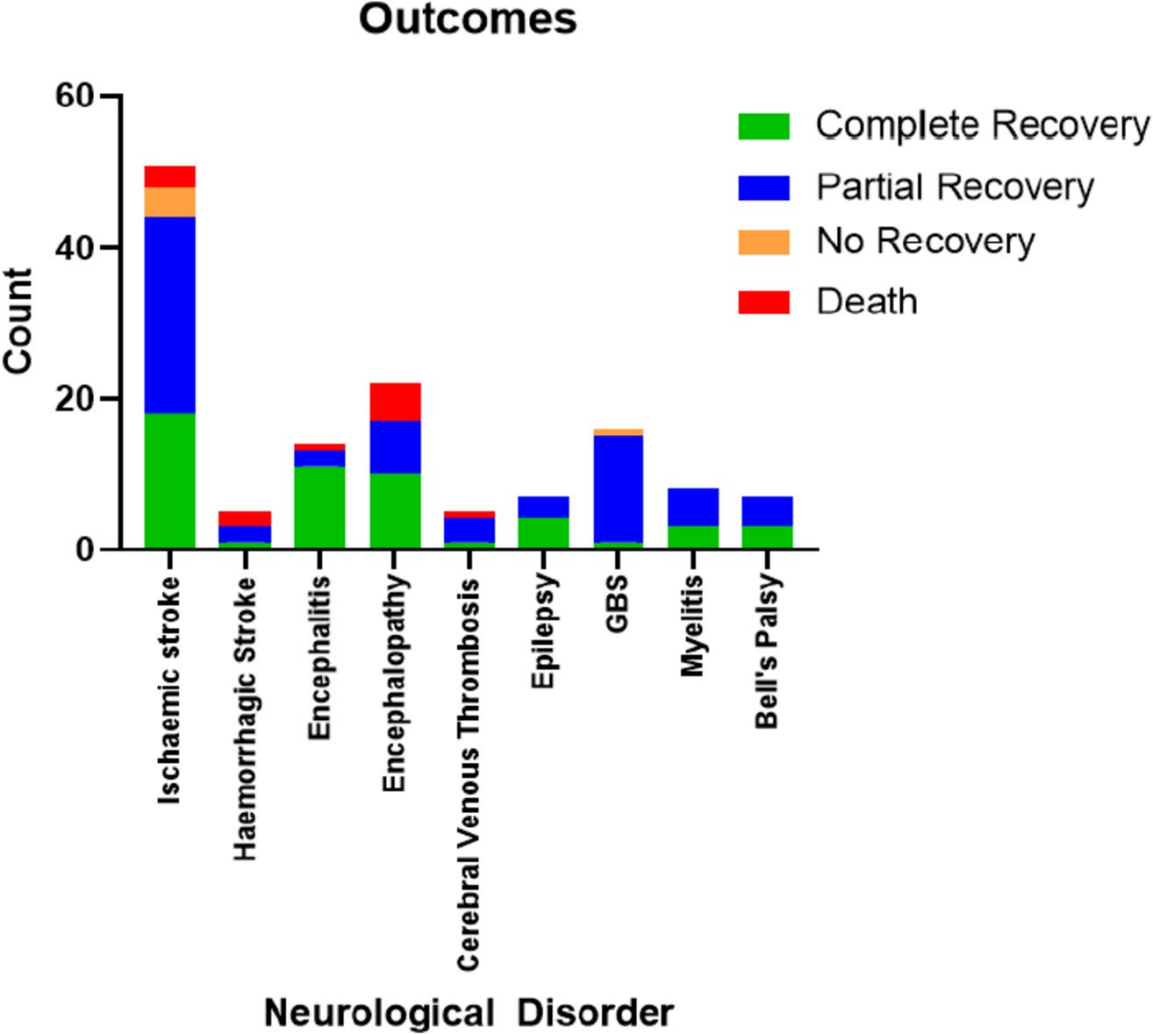
Outcomes of the key neurological disorders associated with COVID-19

Outcomes of the key neurological disorders were categorised as Group A (complete or partial recovery) or Group B (no recovery or death) (Fig. 5) (Table 2). A poor outcome of no recovery or death occurred in seven (13.7%) patients with ischaemic stroke, five (22.7%) patients with encephalopathy, two (40%) patients with haemorrhagic stroke, one (7.1%) patient with encephalitis, one (20%) patient with cerebral venous thrombosis and one (25%) patient with a seizure disorder.

**Table 2:** Key determinants of prognosis of COVID-19 associated neurological disorders. Bold text indicates subsection headings or statistical significance. *p < 0.05 (SD - standard deviation, OR - odds ratio, CI - confidence interval)

Determinant	Group A: Partial/complete recovery (*n* = 144)	Group B: No recovery/death (*n* = 24)	Significance
**Demographic characteristic / comorbidity**			
Age (Mean years)	48.3 (SD 23.8)	56.5 (SD 20.8)	*p* = 0.115
Gender (% male)	53.5% (77)	58.3% (14)	*p* = 0.825
Vaccinated	48.6% (70)	37.5% (9)	*p* = 0.380
Time from last vaccine to neurological onset (mean days)	43.3 (SD 45.5) (*n* = 57)	18.8 (SD 18.2) (*n* = 7)	*p* = 0.166
Symptomatic COVID-19 infection	70.6% (96/136)	75.0% (18/24)	p = 0.808
Supplemental oxygen required	21.5% (31)	45.8% (11)	*p* = 0.020* **OR = 12.93** **(CI = 1.05–160.17; *p* = 0.046)**
Diabetes	21.5% (31)	41.7% (10)	*p* = 0.042*
Hypertension	27.1% (39)	45.8 (11)	*p* = 0.090
Ischaemic heart disease	7.6% (11)	12.5% (3)	*p* = 0.426
**Neurological disorder**			
Ischaemic stroke	86.3% (44/51)	13.7% (7/51)	*p* = 1.000
Haemorrhagic stroke	60.0% (3/5)	40.0% (2/5)	*p* = 0.149
Encephalitis	92.9% (13/14)	7.1% (1/14)	*p* = 0.695
Encephalopathy	77.3% (17/22)	22.7% (5/22)	*p* = 0.322
Cerebral venous thrombosis	80.0% (4/5)	20.0% (1/5)	*p* = 0.542
Epilepsy	100.0 (7/7)	0.0% (0/7)	
Guillain–Barre syndrome	93.8% (15/16)	6.3% (1/16)	*p* = 0.474
Myelitis	100.0% (8/8)	0.0% (0/8)	

Simple comparison analysis was performed to investigate differences in key demographic, comorbidity and disease-related factors between the two groups (Table 2). Group B had a significantly higher percentage of patients with a history of diabetes (*p* = 0.042) and of patients who required supplemental oxygen therapy (*p* = 0.020).

Binary logistic regression analysis was performed with the outcome group as the dependent variable, and all variables with cases in both outcome groups and with significance of *p* < 0.20 on simple comparison analysis, as covariates. Only the requirement of supplemental oxygen was significantly associated with a poor outcome (Group B) (OR: 12.93; *p* = 0.046) (Table 2).

## Discussion

This is the first descriptive study on neurological disorders associated with COVID-19 in Sri Lanka. In this study, we found strokes and encephalopathy to be the most common neurological disorders associated with COVID-19. Distinct patterns of neurological disorders in relation to the COVID-19 variant were observed. This included a high proportion of children presenting with encephalitis and encephalopathy during the Omicron predominant period. There was significant diversity in the time of onset of different neurological disorders in relation to the onset of typical respiratory/systemic symptoms of COVID-19: with ischaemic stroke, encephalitis and encephalopathy occurring within the first week, while GBS, myelitis and Bell palsy had a more delayed onset. The most significant predictor of a poor outcome in our cohort was the need for supplemental oxygen during hospitalisation.

### Spectrum of neurological disorders associated with COVID-19

In our study, strokes accounted for 36.4% while encephalopathy accounted for 13.5% of the neurological disorders associated with COVID-19, similar to reports from other countries [5, 10–13]. The age and sex distribution of neurological disorders in our study was no different to that of non-COVID-19 patients. For example, ischaemic strokes were predominant in males (56.1%) and those over 60 years of age (68.4%) whereas cerebral venous thrombosis was seen predominantly in females (80%) [14, 15].

The next most common COVID-19 associated neurological disorders were inflammatory or immune-mediated disorders of the central (13.0%) and the peripheral (16.8%) nervous systems, with immune-mediated encephalitis and GBS being the commonest (Fig. 2). Our findings were consistent with reports from other parts of the world, as reported in a systematic review of neuroimmune disorders in COVID-19, which identified encephalitis (32.3%, *N* = 133) and GBS (42.9%, *N* = 133) as the most frequent immune mediated neurological conditions [6]. However, curiously, we had only a single case of ADEM in contrast to the high prevalence reported in other regions [16, 17].

Although the frequencies of epilepsy (4.3%) and seizures (2.2%) were similar to studies from other regions in the world (5.7%-10.0%) [5, 12, 18–20], the proportion of patients with movement disorders (2.2%) in our study was lower than that of other studies from Europe (9.3%) [20].

In our paediatric cohort, encephalitis, encephalopathy and GBS were the commonest neurological disorders associated with COVID-19, comparable to reports from other populations [21, 22]. Children presenting with Multisystem Inflammatory Syndrome of COVID (MIS-C) have been found to have a higher frequency of neurological complications [23]. However, none of the children in our study were reported as having developed MIS-C.

A review of COVID-19 associated neurological complications in pregnancy found reports of GBS, posterior reversible encephalopathy syndrome (PRES), cerebrovascular events and encephalopathy [24]. The neurological disorders in the three pregnant patients in our study included haemorrhagic stroke, encephalitis and epilepsy.

### COVID-19 variants and neurological disorders

During the period of our study, the predominant COVID-19 variant in Sri Lanka transitioned from Alpha to Delta (AY.28) to Omicron (BA.1 and BA.2) [9]. Vascular disorders including ischaemic and haemorrhagic stroke and cerebral venous thrombosis were most common during the Delta predominant period while encephalitis, encephalopathy and Bell palsy were most frequent during the Omicron predominant period. The increased frequency of children with encephalitis and encephalopathy during the Omicron predominant period observed in our study is consistent with reports from other regions [25–27]. It has been suggested that the predisposition to COVID-19 related encephalitis in children, particularly with the Omicron variant, may be related to the development of specific mutations in the SARS-CoV-2 spike protein in Omicron subvariants and to exaggerated immune and cytokine responses [26, 27].

### Time of onset of neurological disorders in COVID-19

The majority of cases of ischaemic stroke, encephalitis and encephalopathy occurred at or soon after the onset of COVID-19 symptoms. This may reflect the pathological processes thought to occur in the acute phase of COVID-19 including vascular/thrombotic pathology, blood-brain barrier abnormalities with endotheliopathy, possible viral invasion, an acute innate immune response and the cytokine storm [6, 28]. In contrast, immune-mediated disorders occurred later suggesting different pathogenic phases contributing towards different neurological complications at different stages of the disease course – e.g., an early predominantly vascular/thrombotic phase and a later predominantly inflammatory phase. The observation of encephalitis occurring within the first five days from COVID-19 symptom onset in our study suggests pathology related to direct viral invasion into neuronal cells as observed in other cohorts where encephalitis occurred even before the onset of respiratory symptoms [6]. Rarely, immune-mediated post-COVID encephalitis may occur more than three weeks after pulmonary disease [29].

### Outcomes and associated factors

A majority (73.9%) of the neurological cases reported in our study had symptomatic COVID-19. COVID pneumonia was reported in almost 47% of the symptomatic patients. In our cohort, 23% required oxygen, while smaller numbers (< 7%) received ICU care or had ventilatory requirements.

A requirement for supplemental oxygen therapy emerged as the most significant factor associated with a poor outcome in our cohort (Table 2). It may be argued that those who required supplemental oxygen therapy were more likely to have had severe COVID-19 disease and therefore were more likely to have had a poor outcome.

Among hospitalised patients, the presence of COVID-19 related neurological disorders itself has been linked to increased mortality [5, 13, 30]. We did not have the requisite data to compare mortality between neurological and non-neurological patients in hospital in this study. However, the mortality of the overall cohort of neurological COVID-19 patients (inpatient and outpatient) in this study (7.7%) was higher than the reported overall case fatality rate of COVID-19 patients in Sri Lanka (2.5%) [31].

The strength of our study is that it captured data from all provinces in Sri Lanka over a period of one year during the evolving pandemic with varying virus variants. The limitations are that data collection was not mandatory and that all patients with neurological manifestations may not have been referred to a neurologist, with some being managed by specialist physicians in charge of COVID treatment centres, resulting in some cases not being reported. The lack of a control cohort in this study makes it impossible to compare and assess potential increased risk/association of these conditions with COVID-19 in Sri Lanka.

## Conclusions

The spectrum of neurological disorders occurring in association with COVID-19 in Sri Lanka was similar to that reported from other regions in the world.

### Supplementary Information

**Additional file 1. **

## Data Availability

The datasets used and/or analysed during the current study are available from the corresponding author on reasonable request.

## Abbreviations

COVID-19: Coronavirus disease 2019
SARS-CoV-2: Severe acute respiratory syndrome coronavirus 2
ADEM: Acute disseminated encephalomyelitis
GBS: Guillain-Barre syndrome
ASN: Association of Sri Lankan Neurologists
SD: Standard deviation
PCR: Polymerase chain reaction
RAT: Rapid antigen test
CSF: Cerebrospinal fluid
CNS: Central nervous system
ANOVA: Analysis of variance
OR: Odds ratio
MIS-C: Multisystem inflammatory syndrome of COVID
PRES: Posterior reversible encephalopathy syndrome

## References

[1] WHO Coronavirus (COVID-19) Dashboard. Available from: https://covid19.who.int/. Accessed Feb 2023.

[2] Ellul MA, Benjamin L, Singh B, Lant S, Michael BD, Easton A (2020). Neurological associations of COVID-19. Lancet Neurol.

[3] Yassin A, Nawaiseh M, Shaban A, Alsherbini K, El-Salem K, Soudah O (2021). Neurological manifestations and complications of coronavirus disease 2019 (COVID-19): a systematic review and meta-analysis. BMC Neurol.

[4] Varatharaj A, Thomas N, Ellul MA, Davies NWS, Pollak TA, Tenorio EL (2020). Neurological and neuropsychiatric complications of COVID-19 in 153 patients: a UK-wide surveillance study. Lancet Psychiatry.

[5] Chou SHY, Beghi E, Helbok R, Moro E, Sampson J, Altamirano V (2021). Global incidence of neurological manifestations among patients hospitalized with COVID-19-a report for the GCS-NeuroCOVID consortium and the ENERGY consortium. JAMA Netw Open.

[6] Ariño H, Heartshorne R, Michael BD, Nicholson TR, Vincent A, Pollak TA (2022). Neuroimmune disorders in COVID-19. J Neurol.

[7] Epidemiology Unit COVID-19 situation reports. Available from: https://www.epid.gov.lk/web/index.php. Accessed Feb 2023.

[8] University of Sri Jayewardenepura news report. 2022. Available from: https://www.sjp.ac.lk/covid-19/82-new-omicron-cases-recorded-from-sri-lanka-latest-sars-cov-2-variant-report-by-department-of-immunology-and-molecular-medicine/. Accessed Feb 2023.

[9] Ranasinghe D, Jayathilaka D, Jeewandara C, Gunasinghe D, Ariyaratne D, Jayadas TTP (2022). Molecular Epidemiology of AY.28 and AY.104 Delta Sub-lineages in Sri Lanka. Front Public Heal.

[10] Nannoni S, de Groot R, Bell S, Markus HS (2021). Stroke in COVID-19: A systematic review and meta-analysis. Int J Stroke.

[11] Misra S, Kolappa K, Prasad M, Radhakrishnan D, Thakur KT, Solomon T (2021). Frequency of neurologic manifestations in COVID-19. Neurology.

[12] Bhansali S, Bagrodia V, Choudhury S, Rahman S, Golder M, Tiwari M (2021). Spectrum of hospitalized NeuroCOVID diagnoses from a tertiary care neurology centre in Eastern India. J Clin Neurosci.

[13] Espiritu AI, Sy CM, Anlacan VM, Jamora RD (2021). Neurology and preclinical neurological studies-COVID-19 outcomes of 10,881 patients: retrospective study of neurological symptoms and associated manifestations (Philippine CORONA Study). J Neural Transm.

[14] Luo W, Liu X, Bao K, Huang C (2022). Ischemic stroke associated with COVID-19: a systematic review and meta-analysis. J Neurol.

[15] Bajko Z, Motataianu A, Stoian A, Barcutean L, Andone S, Maier S (2021). gender differences in risk factor profile and clinical characteristics in 89 consecutive cases of cerebral venous thrombosis. J Clin Med.

[16] Wang Y, Wang Y, Huo L, Li Q, Chen J, Wang H (2022). SARS-CoV-2-associated acute disseminated encephalomyelitis: a systematic review of the literature. J Neurol.

[17] Ross Russell AL, Hardwick M, Jeyanantham A, White LM, Deb S, Burnside G (2021). Spectrum, risk factors and outcomes of neurological and psychiatric complications of COVID-19: a UK-wide cross-sectional surveillance study. Brain Commun.

[18] Delorme C, Houot M, Rosso C, Carvalho S, Nedelec T, Maatoug R (2021). The wide spectrum of COVID-19 neuropsychiatric complications within a multidisciplinary centre. Brain Commun.

[19] Thakur KT, Chu VT, Hughes C, Kim CY, Fleck-Derderian S, Barrett CE (2022). Risk factors for new neurologic diagnoses in hospitalized patients with covid-19: a case-control study in New York City. Neurol Clin Pract.

[20] Beghi E, Moro E, Davidescu EI, Popescu BO, Grosu O, Valzania F, et al. Comparative features and outcomes of major neurological complications of COVID-19. Eur J Neurol. 2023;30(2):413–33. 10.1111/ene.15617. 10.1111/ene.15617 PMC9874573 36314485

[21] Gurlevik SL, Ozsurekci Y, Sağ E, DerinOygar P, Kesici S, Akca ÜK (2022). The difference of the inflammatory milieu in MIS-C and severe COVID-19. Pediatr Res.

[22] Fink EL, Robertson CL, Wainwright MS, Roa JD, Lovett ME, Stulce C (2022). Prevalence and risk factors of neurologic manifestations in hospitalized children diagnosed with acute SARS-CoV-2 or MIS-C. Pediatr Neurol.

[23] Valderas C, Méndez G, Echeverría A, Suarez N, Julio K, Sandoval F (2022). COVID-19 and neurologic manifestations: a synthesis from the child neurologist’s corner. World J Pediatr.

[24] Magalhães JE, Sampaio-Rocha-Filho PA (2022). Pregnancy and neurologic complications of COVID-19: A scoping review. Acta Neurol Scand.

[25] Tetsuhara K, Akamine S, Matsubara Y, Fujii S, Kashimada W, Marutani K (2022). Severe encephalopathy associated with SARS-CoV-2 Omicron BA.1 variant infection in a neonate. Brain Dev..

[26] Thongsing A, Eizadkhah D, Fields C, Ballaban-Gil K (2022). Provoked seizures and status epilepticus in a pediatric population with COVID-19 disease. Epilepsia.

[27] Chen CS, Chang CN, Hu CF, Jian MJ, Chung HY, Chang CK (2022). Critical pediatric neurological illness associated with COVID-19 (Omicron BA.2.3.7 variant) infection in Taiwan: immunological assessment and viral genome analysis in tertiary medical center. Int J Infect Dis.

[28] Balcom EF, Nath A, Power C (2021). Acute and chronic neurological disorders in COVID-19: potential mechanisms of disease. Brain.

[29] Gunawardhana C, Nanayakkara G, Gamage D, Withanage I, Bandara M, Siriwimala C (2021). Delayed presentation of postinfectious encephalitis associated with SARS-CoV-2 infection: a case report. Neurol Sci.

[30] Cho SM, White N, Premraj L, Battaglini D, Fanning J, Suen J, et al. Neurological manifestations of COVID-19 in adults and children. Brain. 2023;146(4):1648–61. 10.1093/brain/awac332. 10.1093/brain/awac332 PMC9494397 36087305

[31] Epidemiology unit, Ministry of Health, COVID-19 confirmed deaths. Available from: https://www.epid.gov.lk/web/index.php?option=com_content&view=article&id=233&lang=en. Accessed Feb 2023.

