# Supplementary material for: Neurological disorders associated with COVID-19 in Sri Lanka
Source: BMC Neurol. 2023 Oct 4;23:351. doi: 10.1186/s12883-023-03399-w (PMC10548601; doi:10.1186/s12883-023-03399-w)
Supplement: Supplementary file 1 — Additional file 1. [file 12883_2023_3399_MOESM1_ESM.pdf]

ASN NEUROLOGICAL SURVEILLANCE OF COVID-19

Please tick (√) boxes that apply and specify details as available.

Patient demographics

**Name:**		Hospital:
Address:		Ward: BHT or Clinic No.:
Mobile No.:		Date of presentation/admission: dd/mm/yy
Age (years):	Sex: Male ☐ Female ☐	

COVID-19

Diagnosis: RT-PCR positive result ☐ **Date:** dd/mm/yy **Rapid Antigen Test positive result** ☐ Date: dd/mm/yy

Asymptomatic ☐ Symptomatic ☐ **Date of symptom onset:** dd/mm/yy

COVID-19 related features: Anosmia ☐ Ageusia ☐ COVID-19 Pneumonia ☐ Sepsis ☐

Systemic venous thrombosis: DVT ☐ PE ☐ Other ☐ (specify)____________________________

Treatments received: Anti-virals ☐ ______________________ Corticosteroids ☐ Tocilizumab ☐

Anti-platelets ☐ ______________ Anti-coagulants ☐ ____________________ Other ☐ ________________

Respiratory support: Oxygen ☐ NIV/BIPAP ☐ Mechanical Ventilation ☐ ICU Care ☐

Comorbidities

Diabetes ☐ Obesity ☐ Hypertension ☐ IHD ☐ Stroke ☐ Cardiac failure ☐ Chronic pulmonary disease ☐

CKD ☐ Chronic liver disease ☐ Dementia ☐ Malignancy ☐ (specify) ________________ ____________

Other(specify)____

**Smoker** ☐	**Alcohol excess** ☐	**Pregnant** ☐	**Gestation (weeks):**

Immunosuppressant medication Y ☐ N ☐ (specify)____

COVID-19 vaccine

Dose 1: Date- dd/mm/yy; Type ________________________ **Dose 2: Date-** dd/mm/yy; Type _________________________

Adverse events Y ☐ N ☐ (specify): ____

Neurological manifestations (from **1 week** before up to **6 weeks** after confirmation of SARS-CoV-2 infection)

Sections A-J : Please ONLY complete the section(s) relevant to your patient.

(A) Ischaemic stroke ☐ Date of onset: dd/mm/yy

Minimum criteria: Sudden onset focal neurological deficit with evidence of vascular ischaemic aetiology on imaging (e.g. restricted diffusion on MRI DWI, hypoattenuation consistent with infarct on CT).

Minimum criteria fulfilled? Y ☐ N ☐

Likely predominant aetiology: COVID-19 ☐ **Alternative** ☐ (other significant risk factors- e.g. HT, DM, dyslipidaemia, AF, cardiac failure, previous stroke/TIA, smoker)

(B) Haemorrhagic stroke/ICH ☐ Date of onset: dd/mm/y

Minimum criteria: Sudden onset focal neurological deficit with evidence of haemorrhage on imaging.

Minimum criteria fulfilled? Y ☐ N ☐

Likely predominant aetiology: COVID-19 ☐ **Alternative** ☐ (other significant risk factors- e.g. HT, anticoagulants, dual anti-platelet therapy)

(C) Encephalitis ☐ Date of onset: dd/mm/yy

Minimum criteria: Acute or subacute (<4 weeks) alteration in consciousness, cognition, personality or behaviour, persisting for >24 hours (± new onset seizure/movement disorder, focal neurology, fever) with features of encephalitis on MRI, EEG and CSF (WCC>5cells/mm^3).

Minimum criteria fulfilled? Y ☐ N ☐

Likely predominant aetiology: COVID-19 ☐ **Alternative** ☐ (e.g. evidence of HSV, Dengue, JE, Autoimmune encephalitis etc..)

(D) Encephalopathy ☐ Date of onset: dd/mm/yy

Minimum criteria: Acute or subacute (<4 weeks) alteration in consciousness, cognition, personality or behaviour, persisting for >24 hours, with no evidence of CNS inflammation on MRI or CSF and absence of an alternative diagnosis.

Minimum criteria fulfilled? Y ☐ N ☐

Likely predominant aetiology: COVID-19 ☐ **Alternative** ☐ (e.g. evidence of metabolic, toxic, systemic infective cause)

(E) ADEM (Acute Disseminated Encephalomyelitis) ☐ Date of onset: dd/mm/yy

Minimum criteria: Multifocal clinical CNS events with alteration in consciousness or behaviour AND abnormal brain MRI with typical diffuse, poorly demarcated inflammatory lesions >1cm.

Minimum criteria fulfilled? Y ☐ N ☐

Likely predominant aetiology: COVID-19 ☐ **Alternative** ☐ (evidence of other infections/triggers closely associated with ADEM – e.g. Influenza, Measles, Mumps, Rubella, VZV, EBV, CMV, HSV)

(F) Cerebral venous thrombosis (CVT) ☐	Date of onset: dd/mm/yy

Minimum criteria: Relevant clinical features (e.g. headache, visual disturbance, focal neurological deficits, impaired consciousness, seizures, papilloedema) with evidence of cerebral venous sinus or cortical vein thrombosis on neuroimaging (CT/MR venogram).

Minimum criteria fulfilled? Y☐ N☐
Likely predominant aetiology: COVID-19 ☐ Alternative ☐ (evidence of established causes for CVT- e.g. thrombophilia, OCP, pregnancy, malignancy, head/neck infection)

(G) Epilepsy ☐	Date of onset: dd/mm/yy

Minimum criteria: At least two unprovoked (or reflex) seizures occurring >24 h apart OR one unprovoked (or reflex) seizure and a probability of further seizures of at least 60%, occurring over the next 10 years (ILAE).

Minimum criteria fulfilled? Y☐ N☐
Likely predominant aetiology: COVID-19 ☐ Alternative ☐ (other significant risk factors for epilepsy-e.g. head injury, stroke, dementia, structural/vascular lesions, family history)

(H) Guillain-Barre Syndrome ☐	Date of onset: dd/mm/yy

Minimum criteria: Ascending flaccid paralysis AND global hyporeflexia/areflexia AND monophasic illness pattern with weakness nadir between 12 hours and 28 days. CSF protein level elevated AND CSF total white cell count < 50 cells/mm^3 OR If CSF results unavailable, electrophysiological findings consistent with GBS.

Minimum criteria fulfilled? Y☐ N☐
Likely predominant aetiology: COVID-19 ☐ Alternative ☐ (evidence of other infections/triggers closely associated with GBS- e.g. Campylobacter, Influenza, CMV, EBV, Dengue, HIV, Mycoplasma)

(I) Myelitis ☐	Date of onset: dd/mm/yy

Minimum criteria: Weakness or sensory disturbance of lower ± upper limbs, developing to its worst severity 4h - 21days following onset WITH UMN signs/ sphincter dysfunction/ a sensory level AND absence of a compressive aetiology or spinal AVM on MRI.

Minimum criteria fulfilled? Y☐ N☐
Likely predominant aetiology: COVID-19 ☐ Alternative ☐ (evidence of other established causes/triggers of myelitis – e.g. MS, NMOSD, infections (HSV, VZV, HIV, CMV, EBV, Dengue, TB, Mycoplasma, Lyme, etc.)

(J) Other Neurological diagnosis ☐	Date of onset: dd/mm/yy

Specify details: ____

Likely predominant aetiology: COVID-19 ☐ Alternative☐____

Details of relevant investigations

Investigation	Findings
Blood tests ☐	WBC ________ Platelets________ CRP__________ ESR __________
Lumbar puncture ☐	Opening pressure_____ cm water Neutrophils_____ Lymphocytes ____<br>CSF Protein______ CSF Glucose______ Blood Glucose_____<br>Other (e.g. Viral PCR…)_____________________
Neuroimaging: CT ☐ MRI ☐<br>Angiography ☐ Other☐(specify)_____	
EEG ☐	
Nerve conduction studies/EMG ☐	
Other (specify)__________________	

Treatment for Neurological disease

Corticosteroids ☐ IVIg ☐ Plasma exchange ☐ Other immunosuppressants ☐ __________________________________
Antibiotics☐ ____________________________________ **Antivirals ☐** ____________________________________
Antiplatelets ☐ ___________ **Anti-coagulation ☐** _____________ **Thrombolysis ☐ Thrombectomy☐**

Outcomes

Complete recovery ☐ Partial recovery ☐ No recovery ☐ Death ☐

Reporting source

Name of notifying Consultant:
Ward: Institution: Signature: Date: dd/mm/yy
